# Global public awareness of Castleman disease and TAFRO syndrome between 2015 and 2021: A Google Trends analysis

**DOI:** 10.1002/jha2.459

**Published:** 2022-04-28

**Authors:** Yoshito Nishimura, Midori Filiz Nishimura, David C. Fajgenbaum, Frits van Rhee, Yasuharu Sato, Fumio Otsuka

**Affiliations:** ^1^ Department of Medicine John A. Burns School of Medicine University of Hawai'i Honolulu Hawaii USA; ^2^ Department of General Medicine Okayama University Graduate School of Medicine, Dentistry and Pharmaceutical Sciences Okayama Japan; ^3^ Department of Pathology Okayama University Graduate School of Medicine, Dentistry, and Pharmaceutical Sciences Okayama Japan; ^4^ Center for Cytokine Storm Treatment and Laboratory Division of Translational Medicine and Human Genetics Perelman School of Medicine University of Pennsylvania Philadelphia Pennsylvania USA; ^5^ Myeloma Center University of Arkansas for Medical Sciences Little Rock Arkansas USA; ^6^ Division of Pathophysiology Okayama University Graduate School of Health Sciences Okayama Japan

**Keywords:** Castleman disease, Google Trends, iMCD‐TAFRO, TAFRO syndrome, trend analysis

## Abstract

Castleman disease (CD) is a rare lymphoproliferative disorder with multiple subtypes. Thrombocytopenia, anasarca, fever, reticulin fibrosis or renal insufficiency, and organomegaly (TAFRO) syndrome can occur in the context of CD. The study evaluated worldwide public awareness of CD and TAFRO syndrome using Google Trends data between 2015 and 2021. Our results showed that global public interest steadily grew until late 2019, at a small but significant rate of 1.1% per month from the 1st to 57th month (1/2015–9/2019). The increase coincided with a peak in the United States and Japan, but the search volume decreased at a rate of 1.3% per month after that time. No clear trend changes were noted throughout the study period with the search term “TAFRO.” However, the search volume significantly increased during the time period at a rate of 4.8% (confidence interval [CI]: 2.8, 6.8) and 4.7% (CI: 2.7, 6.8) per month in Japan and worldwide, respectively. There was an insufficient search volume for “TAFRO” in the United States to perform the analysis. Most searches on “TAFRO” stemmed from Japan, suggesting considerable geographical disparity in the awareness of TAFRO syndrome. Further efforts are crucial to raise the awareness of CD and TAFRO syndrome among physicians and the general public, primarily in non‐USA and Japanese countries.

## 1 INTRODUCTION

Castleman disease (CD) is a rare lymphoproliferative disorder with multiple subtypes demonstrating characteristic histopathological features but a range of clinical symptoms [1]. While it is rare and not commonly known, since its first description in 1954 as isolated mediastinal lymphadenopathy, the disease has attracted the attention of clinicians and researchers. In particular, idiopathic multicentric CD (iMCD), defined by negative human herpesvirus 8 and human immunodeficiency virus status, can be life‐threatening with severe systemic inflammation and multiorgan dysfunction. Given the severity and its relatively limited understanding, the Castleman Disease Collaborative Network (CDCN) was founded in 2012 to facilitate CD‐related research and collaboration with various stakeholders, improve awareness and fundraising, and support the patient community [2]. With the efforts of the CDCN, considerable progress has been made over the last several years, including the establishment of the first international consensus diagnostic criteria for iMCD in 2017 [3].

During a similar period, a series of cases with thrombocytopenia (T), anasarca (A), fever (F), reticulin fibrosis or renal insufficiency (R), and organomegaly (O), later described as thrombocytopenia, anasarca, fever, reticulin fibrosis or renal insufficiency, and organomegaly (TAFRO) syndrome, were first reported in 2010 in Japan [4]. TAFRO syndrome is a heterogeneous entity with a constellation of symptoms due to various underlying etiology, including iMCD. iMCD can be separated into iMCD with TAFRO syndrome (iMCD‐TAFRO) and iMCD patients who do not have TAFRO syndrome, which is referred to as iMCD‐not otherwise specified (iMCD) [5]. Since the first diagnostic criteria of TAFRO syndrome were proposed in 2015 [6], a number of cases have been reported all over the world. In fact, over half of the iMCD patients in the CDCN's US‐based ACCELERATE natural history registry have iMCD‐TAFRO [5]. In 2018, the Japanese Ministry of Health, Labour and Welfare established a dedicated research group for CD, TAFRO syndrome, and related diseases to accelerate research and facilitate collaboration with patient groups and other stakeholders [7]. However, no studies have evaluated the public awareness of CD and TAFRO syndrome. Since Internet search is one of the leading sources for health‐related information, online‐based health information‐seeking behavior has been utilized as a reliable surrogate of public awareness [8]. Given the fact that the CDCN is the US‐based, and TAFRO syndrome was first proposed in Japan, we hypothesized that public awareness of CD and TAFRO syndrome may be higher than other countries. Thus, we aimed to evaluate public awareness of CD and TAFRO syndrome since 2015 in the United States, Japan, and worldwide with Google search data.

## 2 METHODS

### 2.1 Data source

Google Trends is a data source based on the total Google search data that have been utilized for various research [9]. The data analysis enables us to describe the relative popularity of specific search terms in a particular category (for instance, “health”), place, and time range. The relative popularity is defined as a relative search volume (RSV), with a scale of 0–100 (100 indicating the highest popularity).

### 2.2 Search input and variables

**FIGURE 1:**
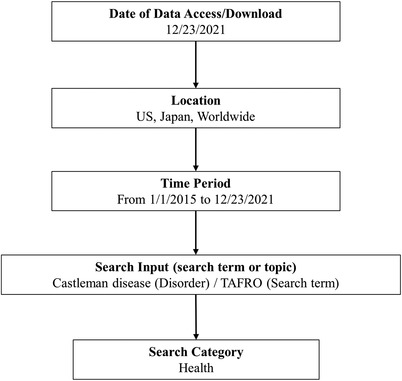
Google Trends search strategy

Our search strategy using Google Trends is summarized in Figure 1 following protocols of previous studies [10, 11]. The search inputs included (CD) with a “Disorder” option, which provides for search volumes of subtopics or relevant themes such as “Castleman‐byo,” a Japanese counterpart of CD, and (TAFRO) with a “Search Term” option. We designated the United States, Japan, and worldwide as the search locations. To exclude the chance that Google search is simply becoming more frequently used, we performed a search with the term (Leukemia), a common hematological disease, with a “Syndrome” option (similar to the “Disorder” option as above). We selected time scales for 84 months (January 2015 to December 2021) to visualize the monthly trends of the RSVs. We also identified the interest of search terms by subregions such as countries, states, or prefectures to see locations where the terms were most popular during the period. Interest by subregion is calculated on a scale of 0–100 (100 indicating that the location has the most popularity as a fraction of total searches in the whole region).

### 2.3 Statistical analyses

The Joinpoint Regression Program (version 4.9.0.0, March 2021, Statistical Research and Applications Branch, National Cancer Institute, USA) was used to analyze a time trend in the Google Trends RSV data [12]. The program enables us to identify time points named joinpoints, where significant temporal trend changes occur. The analysis criteria were set to identify up to three joinpoints. The monthly percent changes (MPCs) between trend‐change points were determined with 95% confidence intervals (CIs). *p*‐value   < 0.05 is defined as the threshold for statistical significance, meaning the level at which the slope differed from zero.

### 2.4 Ethical approval

We used the publicly available data published by Google Trends (Google LLC, Mountain View CA, USA). This study was approved by the Institutional Review Board of Okayama University Hospital with a waiver for informed consent as the study intended to analyze open data retrospectively (No. 1910‐009).

## 3 RESULTS

### 3.1 Trends in the search volume of the term “CD” and “TAFRO”

**FIGURE 2:**
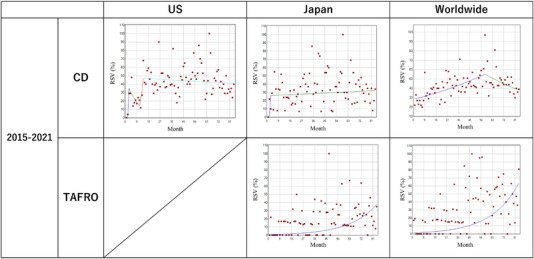
Trends in the monthly relative search volume of “Castleman disease” and thrombocytopenia, anasarca, fever, reticulin fibrosis or renal insufficiency, and organomegaly (“TAFRO”) in the United States, Japan, and worldwide (2015–2021). Monthly relative search volume (RSV) for the search term “Castleman disease” and “TAFRO” is described. The number of slopes is determined by the number of joinpoints identified by the analysis. Joinpoints are the time points when statistically significant changes in the linear slopes are noted

**FIGURE 3:**
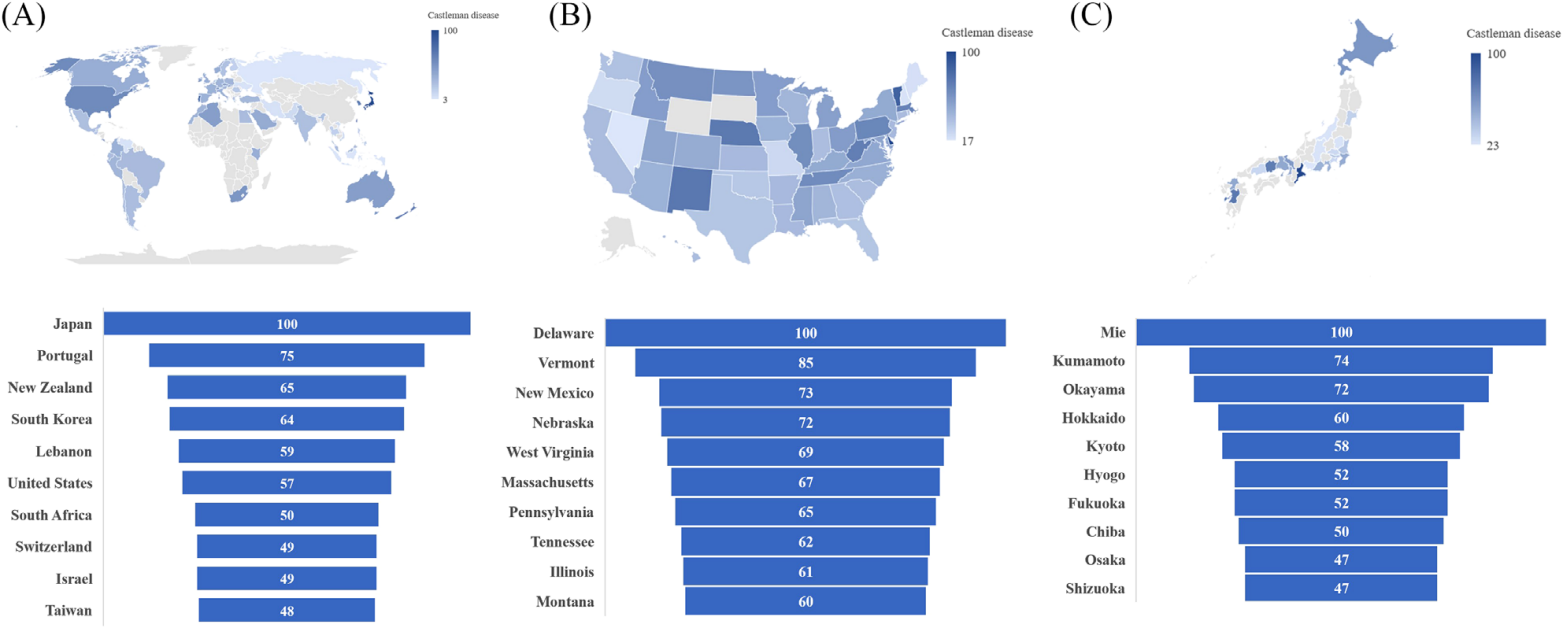
Interest for “Castleman disease” by subregions (2015–2021). (A) Worldwide subregional analysis. (B) Subregional analysis in the United States by state. (C) Subregional analysis in Japan by prefecture

Table 1 and Figure 2 describe trends and trend changes of the monthly RSVs for the search term “CD” and “TAFRO” in the United States, Japan, and worldwide from 2015 to 2021. The first time point in the United States and Japan had a very low volume of searches but there were significant increases in the RSV from the 1st to 3rd month in the United States and Japan up to 1508.6% per month though the search volume was still relatively low. No joinpoints were observed after the third month in Japan. Worldwide, the search volume began at a relatively higher level than the United States or Japan but only increased at a small but significant rate of 1.1% per month from the first to 57th month (January 2015–September 2019), with a decrease of the RSV of 1.3% after that time. Over the course of the time range, there were significant increases in average MPCs of 7.1% (CI: 2.1, 11.8) and 5.9% (CI: 1.6, 10.3) in the United States and Japan, respectively (Table 2). In contrast, the average MPC worldwide did not increase significantly over the course of the time range (mean: 0.3%, CI: −0.1, 0.7). In subregional analyses (Figure 3), relative interest for “CD” was the highest in Japan. Portugal, New Zealand, South Korea, Lebanon, and the United States were the next five top Google searchers worldwide. In the United States and Japan, interest in CD is widely distributed throughout their subregions.

**TABLE 1:** Trend changes in relative search volumes of “Castleman disease” and “TAFRO” (2015–2021)

		Period 1		Period 2		Period 3		Period 4	
Word	Country	Months	MPC (%) (95% CI)	Months	MPC (%) (95% CI)	Months	MPC (%) (95% CI)	Months	MPC (%) (95% CI)
Castleman disease	USA	1/2015–3/2015	1508.6* (520.8, 4067.8)	3/2015–11/2015	−10.3 (−21.0, 1.8)	11/2015–2/2016	47.4 (−43.1, 281.9)	2/2016–12/2021	−0.2 (−0.6, 0.2)
	Japan	1/2015–3/2015	862.3* (71, 5315.6)	3/2015–12/2021	0.3 (−0.3, 0.8)				
	Worldwide	1/2015–9/2019	1.1* (0.8, 1.4)	9/2019–12/2021	−1.3* (−2.3, −0.3)				
TAFRO	Japan	1/2015–12/2021	4.8* (2.8, 6.8)						
	Worldwide	1/2015–12/2021	4.7* (2.7, 6.8)						

*Note*: Periods were separated as Period 1–4, when the trend changes were statistically detected in the Joinpoint regression analysis during the study period. Monthly percentage changes are shown from month 1 (January 2015) to month 84 (December 2021). There was an insufficient search volume for “TAFRO” in the United States to perform the analysis.

Abbreviations: CI, confidence interval; MPC, monthly percentage change; TAFRO, thrombocytopenia, anasarca, fever, reticulin fibrosis or renal insufficiency, and organomegaly.

* Significantly different from zero (*p* < 0.05).

**TABLE 2:** Average monthly trends in relative search volumes of relevant search terms (2015–2021)

Word	Country	Average MPC (%) (95% CI)
Castleman disease	USA	7.1* (2.7, 11.8)
	Japan	5.9* (1.6, 10.3)
	Worldwide	0.3 (−0.1, 0.7)
TAFRO	Japan	4.8* (2.8, 6.8)
	Worldwide	4.7* (2.7, 6.8)

*Note*: Average monthly percentage changes are shown from month 1 (January 2015) to month 84 (December 2021).

Abbreviations: CI, confidence interval; MPC, monthly percentage change; TAFRO, thrombocytopenia, anasarca, fever, reticulin fibrosis or renal insufficiency, and organomegaly.

* Significantly different from zero (*p* < 0.05).

**FIGURE 4:**
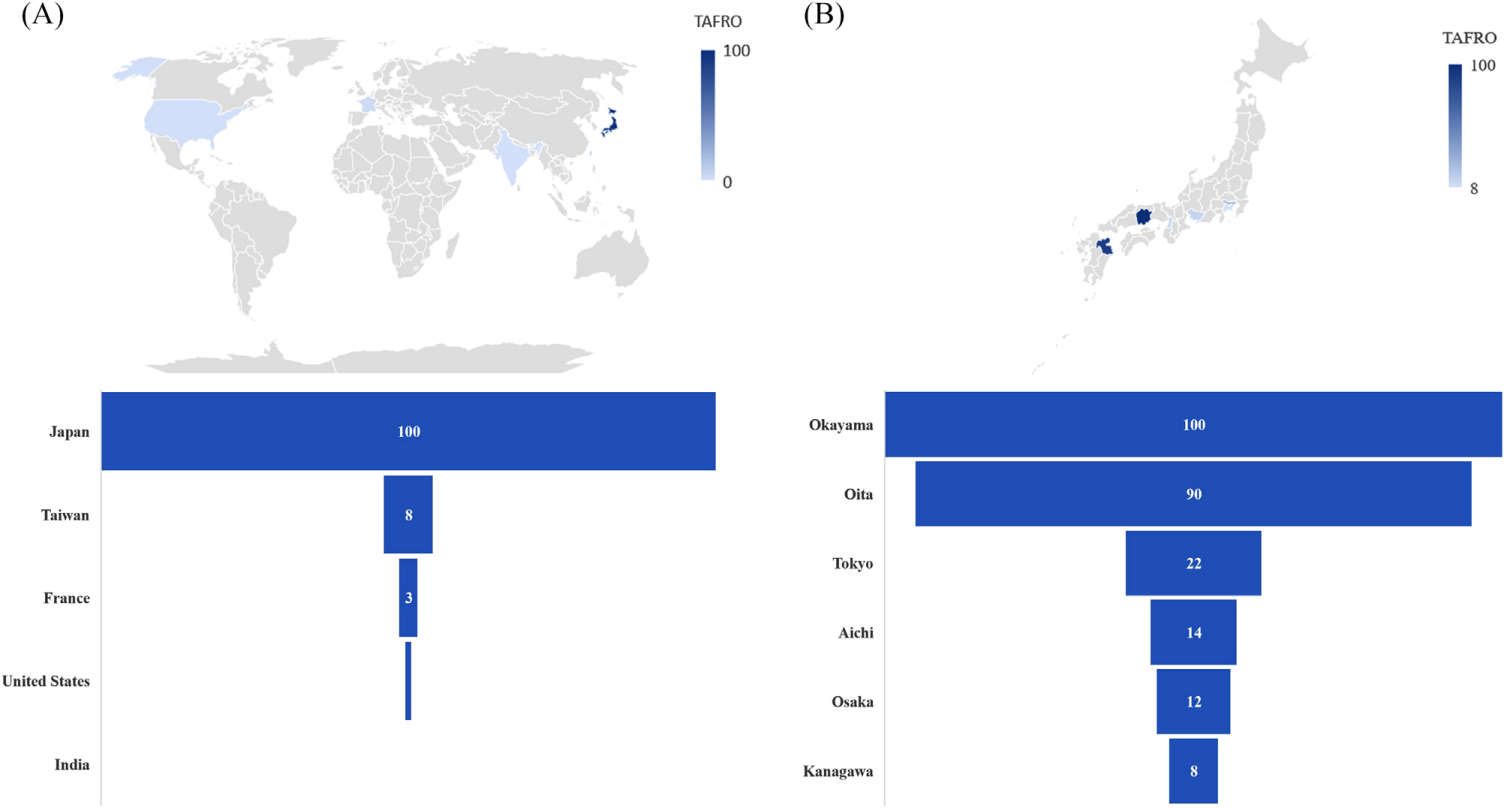
Interest for thrombocytopenia, anasarca, fever, reticulin fibrosis or renal insufficiency, and organomegaly (“TAFRO”) by subregions (2015–2021). (A) Worldwide subregional analysis. (B) Subregional analysis in Japan by prefecture

Regarding the search term “TAFRO,” no clear trend changes were noted throughout the study period. The average MPCs significantly increased during the time period at a rate of 4.8% (CI: 2.8, 6.8) and 4.7% (CI: 2.7, 6.8) in Japan and worldwide, respectively. There was an insufficient search volume for “TAFRO” in the United States to perform the analysis. In subregional analysis (Figure 4), interest for “TAFRO” was disproportionately distributed worldwide, with almost all the searches stemming from Japan. In Japan, most of the searches were from Okayama and Oita prefectures.

### 3.2 Trends in the search volume of the term “leukemia”

Figures S1 and S2 describe our search method, trends, and trend changes of the monthly RSVs for the search term “leukemia” in the United States, Japan, and worldwide from 2015 to 2021. While there were several joinpoints in search results in the United States and worldwide, the average MPCs in the United States, Japan, and worldwide were 0 (CI: −0.5–0.5), 0.1 (CI: −0.2–0.4), and 0 (CI: −0.6–0.5), respectively.

## 4 DISCUSSION

This study evaluated the extent of public awareness for CD and TAFRO syndrome using the RSV of Google Trends data as a surrogate. This is the first study to assess public awareness regarding CD and TAFRO syndrome. Our results showed that the public interest in CD significantly increased in the United States and Japan in early 2015 from a very low level, and the extent of interest remained at a similar level after that. Given the average MPCs with the term “leukemia” during the period as a contrasting search were almost zero, the increase may be attributable to an increase in public awareness, not to the popularity of Google search. A similar trend was observed worldwide, although interest grew at a more graduate rate from early 2015 until late 2019, and there has been a gradual decline over the last 2 years. Although public interest toward TAFRO syndrome has been trending up in Japan and worldwide, the extent of increase remains scarce, and there was a considerable geographical disparity in the public interest toward “TAFRO,” with most of the searches coming from two prefectures in Japan. The results suggest that further efforts are important to increase awareness for CD and TAFRO syndrome. In particular, TAFRO syndrome or iMCD‐TAFRO has remained relatively unknown outside of certain regions of Japan and academic medical centers treating iMCD patients worldwide.

There are several events and campaigns that can explain some of the top spikes in Google searches. The top three spikes in “CD” searches in the United States and worldwide all corresponded to major public awareness and media exposure related to the CDCN and one of its co‐founders. The top spike (100%) in the United States and the second highest spike worldwide occurred during the month (May 2020) that the CDCN co‐founder gave the keynote address for ASCO, and his memoir, Chasing My Cure, became a national bestseller following over 5 M views of a social media video. The second highest spike in the United States occurred during the month (February 2017) that the New York Times ran a cover story about a physician who suffered from CD and his story to find a treatment [13]. The third highest spike in the United States (87%), the top spike in Japan, and the top spike worldwide occurred around the launch of Chasing My Cure (September 2019) [14]. Separate from these spikes, there was also a relative increase in searches over the 6‐year time period. Although these results highlight that media exposure contributes to spikes in interest, organizations like the CDCN work to consistently raise awareness of CD including social media exposure, fundraising activities, the establishment of World Castleman Disease Day on July 23rd, and dissemination of educational material [2]. Given the general plateau of public awareness of CD, additional measures to reach more populations may be needed with a focus on countries other than the United States and Japan, such as the use of multilingual materials in social media or educational materials, collaboration with non‐US/Japanese organizations [15].

The low public awareness of TAFRO syndrome or iMCD‐TAFRO is concerning. Although considerable efforts have been made to establish diagnostic criteria of TAFRO syndrome and an international definition of iMCD‐TAFRO [5, 16], there is limited awareness of these entities even in Japan, and it may remain virtually unknown in other countries, especially in African and non‐Japanese Asian countries. The subregional differences in the RSVs of CD in the United States and Japan may be related to the presence of core hospitals or research institutes working on CD. For example, Pennsylvania in the United States and Okayama in Japan have research centers leading CD‐related research. The disproportionate regional distribution for “TAFRO” in Japan may be explained with the same hypothesis, as Okayama prefecture has a research institute working on TAFRO syndrome and iMCD‐TAFRO. To address the issues with low public awareness and disproportionate distribution of interests, in addition to research activities, a focus on awareness raising is crucial for researchers working on the area.

A strength of our study is that it is the first to quantify the extent of public awareness of CD with Google Trends as the data source. Several limitations need to be addressed. First, the results of Google searches among only those with Internet access and seeking health information with Google search are used as surrogates of disease awareness. However, with the high global Internet penetration rates and Google search market share [17], Google Trends is considered a good surrogate of public awareness and engagement. Another limitation is that the spikes in Google searches corresponded to specific events involving media, which is not the intent of this study. Direct measures such as questionnaires could be beneficial to verify the findings.

In conclusion, this study suggests that public awareness of CD and TAFRO syndrome is limited in non‐US or Japanese countries. In particular, awareness‐raising activities for TAFRO syndrome or iMCD‐TAFRO should be a future area requiring urgent attention.

## FUNDING INFORMATION

The authors received no specific funding for this work.

## CONFLICT OF INTEREST

The authors declare no conflict of interest in association with the present study.

## ETHICS STATEMENT

We used publicly available data published by Google Trends (Google LLC, Mountain View CA, USA). The study was approved by the Institutional Review Board of Okayama University Hospital with a waiver for informed consent (No. 1910‐009). All research methods were performed according to relevant guidelines and regulations.

## AUTHOR CONTRIBUTIONS

YN proposed the study concept, wrote the manuscript, designed the study, and analyzed the data. MFN, DCF, FVR, YS, and FO critically revised the manuscript.

## Supporting information

Supplementary Figure 1. Google Trends search strategy for “leukemia”

Supplementary Figure 2. Trends in the monthly relative search volume of “leukemia” in the United States, Japan, and worldwide (2015–2021). Monthly relative search volume (RSV) for the search term “leukemia” is described. The average monthly percentage changes in the United States, Japan, and worldwide were 0 (confidence interval [CI]: −0.5–0.5), 0.1 (CI: −0.2–0.4), and 0 (CI: −0.6–0.5), respectively.

## Data Availability

The data that support the findings of this study are available from the corresponding author upon reasonable request.
