# Supplementary figures and images for: Global public awareness of Castleman disease and TAFRO syndrome between 2015 and 2021: A Google Trends analysis
Source: EJHaem. 2022 Apr 28;3(3):748–53. doi: 10.1002/jha2.459 (PMC9421978; doi:10.1002/jha2.459)

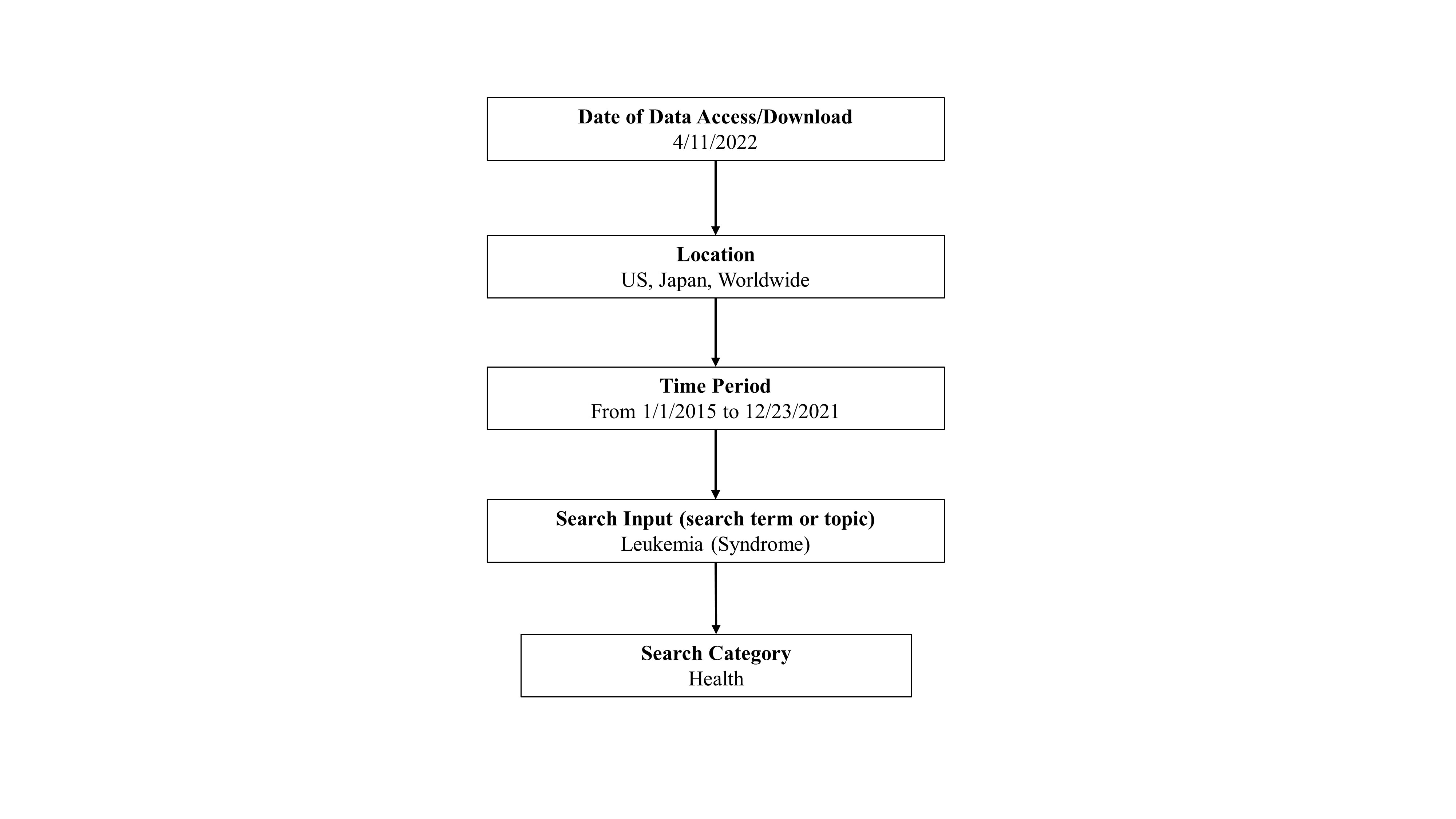

Supplement: Supplementary file 1 — Supplementary Figure 1. Google Trends search strategy for “leukemia” [file JHA2-3-748-s002.JPG]

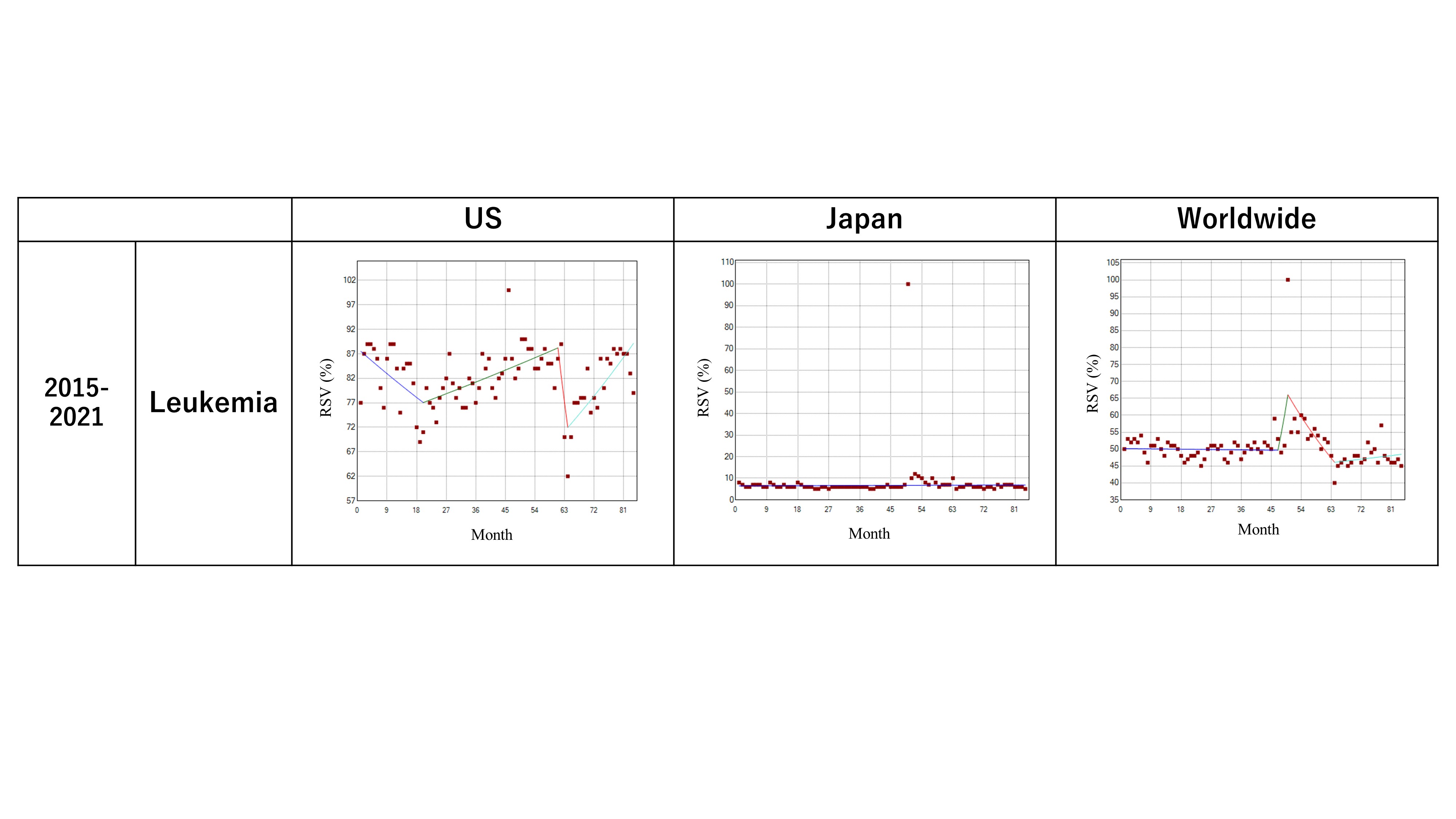

Supplement: Supplementary file 2 — Supplementary Figure 2. Trends in the monthly relative search volume of “leukemia” in the United States, Japan, and worldwide (2015–2021). Monthly relative search volume (RSV) for the search term “leukemia” is described. The average monthly percentage changes in the United States, Japan, and worldwide were 0 (confidence interval [CI]: −0.5–0.5), 0.1 (CI: −0.2–0.4), and 0 (CI: −0.6–0.5), respectively. [file JHA2-3-748-s001.JPG]
